# Microsatellite variability between apricot and related *Prunus* species

**DOI:** 10.1186/1753-6561-5-S7-P10

**Published:** 2011-09-13

**Authors:** Fatemeh Maghuly, Margit Laimer

**Affiliations:** 1 Plant Biotechnology Unit (PBU), Dept. Biotechnology, University of Natural Resources and Life Sciences, Muthgasse 18, 1190 Vienna, Austria

## Background

Apricot*s*, family *Rosaceae*, are economically important representatives of the genus *Prunus.* The number of apricot species ranges from three to ten, depending on the classification system adopted. The major obstacles to expansion of apricot production are irregular yields and low resistance to diseases. Compared with the high genetic variability in related species, apricots are not so variable and thus interspecific hybrids were proposed as a means to overcome deficiencies inherent in the common apricot. To estimate the extent of variation in apricot germplasm, between ecogeographical groups and related species, is very useful for planning breeding programmes, through diversity analysis, cultivar identification or marker-assisted selection.

## Methods

One hundred accessions of *P. armeniaca* representing the European, Irano-Caucasian, Central Asian and North American genepool, three accessions of *P. mandshurica* and one accession each of *P. ansu*, *P. brigantiaca*, the interspecific hybrid Plumcot,*P. x dasycarpa*, *P. mume*, *P. siberica* were analysed with 10 apricot SSR loci on an ABI 3100 capillary sequencer [1]. A total of 196 alleles were detected varying from 11 to 27 with an average of 16.9. Out of them, 59 alleles occurred only oce (so-called private alleles). 34 (58%) private alleles were also found in related species. Non- amplified alleles were observed in four samples of the related species (*P. siberica* and *P.brigantina*). F_ST_ values ranged from 0.5121 to 0.3503, with an average of 0.4459 (Table 1). It is known, that F_ST_ values up to 0.05 indicate negligible genetic differentiation whereas >0.25 means very great genetic differentiation within the population analyzed. In *Prunus*, high levels of genetic differentiation could be explained by the mating system. The data indicate a low gene flow (as described by Nm; Table 1). These results could be attributed to different geographic origins of the species or the influence of the breeding strategy.

**Table 1:** Variability parameters calculated for 10 SSR markers in 100 apricot cultivars origin of five ecogeogrphical region and related species using POPGENE

Locus	Number of putative alleles	Effective alleles per locus(Ne)	Observed heterozygosity (Ho)	Expected heterozygosity (He)	Inbreeding coefficient (F_ST)_	Gene flow (Nm)
SsrPaCITA7	18	4.6280	0.8091	0.7875	0.4252	0.3380
ssrPaCITA10	21	4.4906	0.5545	0.7809	0.4461	0.3105
ssrPaCITA19	14	4.1052	0.7636	0.7599	0.5121	0.2382
ssrPaCITA23	11	4.5846	0.5872	0.7855	0.4951	0.2550
ssrPaCITA27	12	2.9979	0.3738	0.6696	0.6130	0.1578
UDAp-407	27	7.2979	0.7182	0.8669	0.3823	0.4039
UDAp-410	15	5.5415	0.8440	0.8233	0.4493	0.3064
UDAp-414	20	5.3862	0.5000	0.8181	0.3503	0.4636
UDAp-415	15	3.7162	0.6273	0.7342	0.4072	0.3640
UDAp-420	16	3.5567	0.5909	0.7221	0.4102	0.3594
Mean	16.9	4.6305	0.6369	0.7748	0.4459	0.3107
St.Dev	4.77	1.2233	0.1471	0.0565	-	-

## Results and conclusions

**Figure 1:**
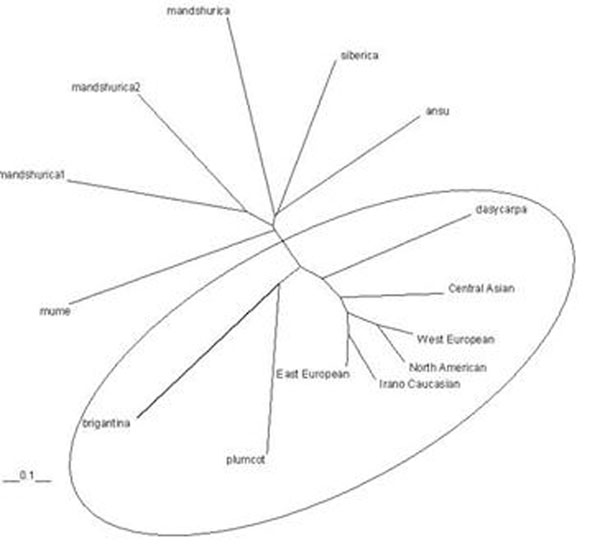
Neighbor joining dendrogram for apricots from five ecogeographical groups and related species.

Genetic similarity among common apricots and related species was quantified using NeiÂ´s [2] genetic distance and genetic identity based on allele frequencies (data not shown). The lowest genetic identity (0.000) was found among *P. mandshurica*, *P. brigantina*, *P.mume* and *P. dasycarpa*, and between Plumcot and *P. mume.* The highest genetic identities (0.81) were found between Western European and North American accesssions and among Irano Caucasian and Eastern European groups. To demonstrate the genetic relationship between common apricots from different ecogeographic regions and related species, a neighbor joining dendrogram based on genetic distance was produced (Figure 1). The accessions are divided into two groups, one containing all common apricots and hybrids thereof, and the other containing all related species. In general, results show that the common apricots are remote from related species. The tree supports that the *P. x dasycarpa* and Plumcot, known to be a *P. armeniaca* hybrid, are intermediates between common apricot and other related species. *P. ansu* and *P. siberica* appear distant from the others accessions, and in fact they are the most distanctly related species. It is interesting to known that some authors even consider *P. ansu* as a separate species [3]. In this study *P. siberica*, the species having the largest distribution area of all apricot species, and *P. mandshurica*, being present in the very cold area, cluster together. In the analyses they appear far from the common apricot species, which confirms them as being markedly different from *P. armeniaca*. Maghuly et al. [1] described that *P. x dasycarpa*, *P. brigantiaca* and Plumcot were distant from the common apricot cluster*.* In fact, *P. brigantiaca* is the most distantly related species, *while P. x dasycarpa*, a hybrid between *P. armeniacax P. cerasifera*, was found intermediate between the apricot groups and Plumcot, which is a hybrid between *P. armeniaca* and *P. salicinia*.

Results to date indicate that crosses between apricot and apricot related species are successful, when made in either direction and the resulting hybrids are viable [3]. In this manner, *P. mandshurica* and *P. siberica* were used in common apricot breeding as a source of cold hardiness [4]. Likewise, adaptation to humid climates should be easy to transmit through hybridization with *P. mume* and *P. armeniaca* var. *ansu*. In addition, Rubio et al. [5] suggested *P. manchurica* as the possible origin of the apricot cultivars resistance to Sharka. It should be emphasiszed that in the interest of still further extending the genetic diversity available for posterity, additional efforts should be committed to the systematic exploration for unique phenotypes of apricots, and also of related species.

## References

[B1] Maghuly F Borroto Fernandez E Ruther S Bistray G Pedryc A Laimer M Microsatellite variability in apricots (Prunus armeniaca L.) reflects their geographic origin and breeding history Tree Genetics and Genomes 2005 1 151 165 10.1007/s11295-005-0018-9

[B2] Nei M Estimation of average heterozygosity and genetic distance from a small number of individuals Genetics 1978 89 583 590 17248844 10.1093/genetics/89.3.583 PMC1213855

[B3] Mehlenbacher SA Cociu V Hough F Moore JN and Ballington RJ Apricot (*Prunus*) Genetic resource of temperate fruit and nut crops 1991 ISHS Wageningen 65 107

[B4] Paunovic SA Apricot germplasm, breeding, selection, cultivar, rootstock and environment Acta Hort 1988 209 13 28

[B5] Rubio M Dicenta F Martínez-Gómez P Susceptibility to sharka (*Plum pox virus*) in*Prunus mandshurica x Prunus armeniaca* seedlings Plant Breeding 2003 122 465 466 10.1046/j.1439-0523.2003.00902.x

